# Sensitivity of ^18^F-fluorodihydrotestosterone PET-CT to count statistics and reconstruction protocol in metastatic castration-resistant prostate cancer

**DOI:** 10.1186/s13550-019-0531-8

**Published:** 2019-07-30

**Authors:** Matthijs C. F. Cysouw, Gerbrand M. Kramer, Dennis Heijtel, Robert C. Schuit, Michael J. Morris, Alfons J. M. van den Eertwegh, Jens Voortman, Otto S. Hoekstra, Daniela E. Oprea-Lager, Ronald Boellaard

**Affiliations:** 1 Amsterdam UMC, Vrije Universiteit Amsterdam, Department of Radiology and Nuclear Medicine, Cancer Center Amsterdam, De Boelelaan 1117, Amsterdam, The Netherlands; 2 Philips Healthcare, Best, The Netherlands; 3 Department of Medicine, Genitourinary Oncology Service, Memorial Sloan Kettering Cancer Center, 353 E 68th St, New York, NY 10065 USA; 4 Amsterdam UMC, Vrije Universiteit Amsterdam, Department of Medical Oncology, Cancer Center Amsterdam, De Boelelaan 1117, Amsterdam, The Netherlands

**Keywords:** [^18^F]-fluordihydrotestosterone, PET-CT, mCRPC, Count statistics, Reconstruction

## Abstract

**Objectives:**

Whole body [^18^F]-fluorodihydrotestosterone positron emission tomography ([^18^F]FDHT PET) imaging directly targets the androgen receptor and is a promising prognostic and predictive biomarker in metastatic castration-resistant cancer (mCRPC). To optimize [^18^F]FDHT PET-CT for diagnostic and response assessment purposes, we assessed how count statistics and reconstruction protocol affect its accuracy, repeatability, and lesion detectability.

**Methods:**

Whole body [^18^F]FDHT PET-CT scans were acquired on an analogue PET-CT on two consecutive days in 14 mCRPC patients harbouring a total of 336 FDHT-avid lesions. Images were acquired at 45 min post-injection of 200 MBq [^18^F]FDHT at 3 min per bed position. List-mode PET data were split on a count-wise basis, yielding two statistically independent scans with each 50% of counts. Images were reconstructed according to current EANM Research Ltd. (EARL1, 4 mm voxel) and novel EARL2 guidelines (4 mm voxel + PSF). Per lesion, we measured SUVpeak, SUVmax, SUVmean, and contrast-to-noise ratio (CNR). SUV was normalized to dose per bodyweight as well as to the parent plasma input curve integral. Variability was assessed with repeatability coefficients (RCs).

**Results:**

Count reduction increased liver coefficient of variation from 9.0 to 12.5% and from 10.8 to 13.2% for EARL1 and EARL2, respectively. SUVs of EARL2 images were 12.0–21.7% higher than EARL1. SUVs of 100% and 50% count data were highly correlated (*R*^2^ > 0.98; slope = 0.97–1.01; ICC = 0.99–1.00). Intrascan variability was volume-dependent, and count reduction resulted in higher intrascan variability for EARL2 than EARL1 images. Intrascan RCs were lowest for SUVmean (8.5–10.6%), intermediate for SUVpeak (12.0–16.0%), and highest for SUVmax (17.8–22.2%). Count reduction increased test-retest variance non-significantly (*p* > 0.05) for all SUV types and normalizations. For SUVpeak at 50% of counts, RCs remained < 30% when small lesions were excluded. Splitting data reduced CNR by median 4.6% (interquartile range 1.2–8.7%) and 4.6% (interquartile range 1.2–8.7%) for EARL1 and EARL2 images, respectively.

**Conclusions:**

Reducing [^18^F]FDHT PET acquisition time from 3 min to 1.5 per bed position resulted in a repeatability of SUVpeak (bodyweight) remaining ≤ 30%, which is generally acceptable for response monitoring purposes. However, EARL2 reconstruction was more affected, especially for SUVmax whose repeatability tended to exceed 30%. Lesion detectability was only slightly impaired by reducing acquisition time, which might not be clinically relevant in mCRPC.

**Electronic supplementary material:**

The online version of this article (10.1186/s13550-019-0531-8) contains supplementary material, which is available to authorized users.

## Introduction

The androgen receptor (AR) axis plays a central role in hormone sensitive as well as castrate-resistant prostate cancer (CRPC) [1]. In the last decades, several AR signalling inhibitor (ARSi) therapies have been developed and approved for the treatment of metastatic (m)CRPC patients [2–5]. Results of the initial treatment with ARSi therapies (e.g. enzalutamide and abiraterone) are excellent, with mild toxicity profiles. Unfortunately, initial treatment response and response durability are variable, and response to second-line ARSi therapies is often short [3, 5]. Therefore, a predictive biomarker for response to these ARSi drugs is urgently needed. Currently used imaging modalities (e.g. CT and bone scintigraphy) for restaging and detection of disease progression in CRPC are not suited for this purpose [6].

^18^F-fluorodihydrotestosterone ([^18^F]FDHT) positron emission tomography-computed tomography (PET-CT) directly targets the AR in whole body imaging [7, 8]. Hereby it can assess AR status on a lesion-by-lesion level allowing for characterization of AR expression and its intrapatient heterogeneity in vivo [9]. This may not only enable prognostication for ARSi therapies, but also facilitate novel AR-targeted drug development [10, 11]. Recently, technical validation studies on the optimal simplified metrics and their repeatability have been performed and clinical studies evaluating the value of ^18^F-FDHT PET/CT as an imaging biomarker in the clinical setting are ongoing [8, 12].

Crucial elements of validation and clinical implementation of novel oncologic PET tracers and their imaging protocols are patient burden and cost of imaging. The latter two should be as low as possible, while maintaining high quantitative and qualitative accuracy for clinical purposes such as prediction or monitoring of treatment response. Until now, whole body [^18^F]FDHT PET/CT studies have been acquired at 3–4 min per bed position, resulting in a typical in-scanner time of about 30 min for a single scan session [7, 12]. As mCPRC patients often have extensive (painful) metastatic disease, frequently involving the spine, reducing acquisition time could diminish patient burden, reduce the cost of imaging, and improve department efficiency. This requires that the effect of count statistics on the performance of [^18^F]FDHT PET-CT is known. For [^18^F]FDG PET-CT, it has been shown that reducing acquisition times may reduce image quality, but does not necessarily affect lesion detection rates [13, 14].

The finite spatial resolution of current PET scanners leads to blurring of images and causes partial-volume effects. Therefore, the EANM Research Ltd. (EARL) group has incorporated the point spread function (PSF) in reconstruction algorithms in the novel EARL2 (2019) guideline to improve image resolution [15, 16]. These novel standards could negatively affect quantitative precision and thereby hamper both prediction and monitoring of treatment response [17, 18]. Also, they could affect comparability of data between centres in multicentre trials. Therefore, it is important to know how these reconstruction protocols and their sensitivity to count statistics affect accuracy and precision of [^18^F]FDHT PET-CT studies.

To technically validate [^18^F]FDHT PET-CT for trials and future clinical use, i.e. for drug development, prognostication, and prediction or monitoring of response, it is crucial to know whether and how accuracy and precision of [^18^F]FDHT PET-CT are a function of image count statistics and reconstruction protocol. Therefore, the aim of this study was to assess how count statistics and reconstruction protocol (EARL1 [2015] vs EARL2 [2019] guidelines) affect accuracy, repeatability, and lesion detectability of analogue whole body [^18^F]FDHT PET-CT.

## Methods

### Patients

Fourteen histologically proven mCRPC patients were prospectively included at the Amsterdam UMC (location VUmc), the Netherlands, between February 2015 and April 2016, as part of a multicentre cohort study [12]. Patient eligibility criteria were as follows: castrate levels of serum testosterone (< 1.7 nmol/L [50 ng/dL]), ≥ 1 month since their last anti-cancer pharmacologic therapy, no concurrent malignancies, and progressive disease based on any of the following: (a) a rise in PSA through 3 consecutive measurements, (b) RECIST 1.1 imaging evidence of progressive disease, and/or (c) bone scan showing at least two new metastatic lesions not attributable to flare phenomenon. Patients without orchiectomy remained on androgen depletion therapy with a gonadotropin-releasing hormone analogue or inhibitor during the study. The Amsterdam UMC (location VUmc) institutional review board approved this prospective study, and each subject gave written informed consent prior to study enrolment.

### PET imaging protocol

Patients were scanned on two consecutive days on a whole body time-of-flight Gemini TF64 PET-CT scanner (Philips Healthcare, Netherlands) with EARL accreditation [16]. A 4-h fasting period was included to minimize intra-intestinal bile activity. Intravenous injection of ± 200 MBq [^18^F]FDHT was followed by a 30-min dynamic scan of the chest (with aorta in the field of view) to acquire an image-derived input function. Venous blood samples were drawn at 5, 10, and 30 min. Analysis of venous samples included measurements of the whole blood and plasma activity concentrations, parent fraction, and metabolites (details in [19]). A whole body scan (3 min/bed position) was made from the mid-thigh to the skull vertex at 45 min post-injection. Complying with the EARL1 guideline [16], whole body PET images were reconstructed with standard iterative time-of-flight reconstruction algorithm (BLOB-OS-TF) with 3 iterations and 33 subsets, with a matrix size of 144 × 144 and voxels 4 × 4 × 4 mm. Images were corrected for scatter, random coincidences, decay, and attenuation (low-dose CT; 80 mA at 120–140 kV, 5-mm slice thickness).

We additionally reconstructed images with the PSF algorithm as provided by the vendor (Philips Healthcare) to conform with EARL2 guidelines. This comprises post-reconstruction image processing using the Richardson-Lucy iterative deconvolution algorithm with sieve noise regularization (PSF option: 1 iteration, regularization full-width-at-half-max at 6 mm) as the resolution recovery method [20]. This algorithm uses a scanner-specific spatially variant PSF to improve image resolution and is described as follows [20]:

1 $$ {I}_{i+1}=\frac{I_i}{f\ast s}\left(f\ast s\ast \frac{I_{\mathrm{o}}}{I_i\otimes s\otimes f}\right) $$

where *I*_*i* + 1_ is the current image estimate, *I*_*i*_ is the image estimate from the *i*th iteration, *f* is the system Gaussian PSF, *s* is the sieve kernel, and *I*_o_ is the original measured image.

**Fig. 1:**
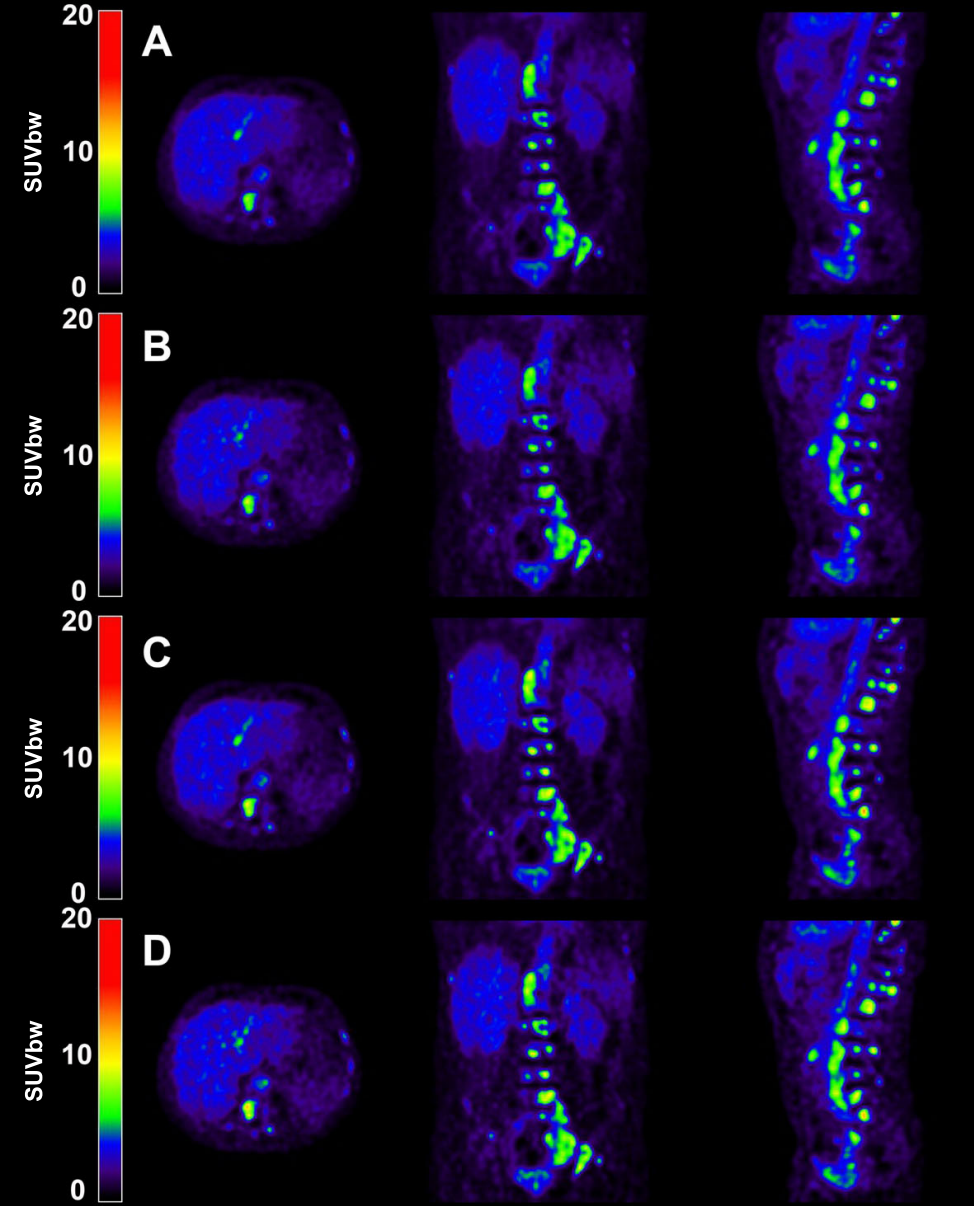
Illustration of a PET image of a typical mCRPC patient with extensive [^18^F]FDHT-avid bone metastases reconstructed with **a** 100% count EARL1, **b** 50% count EARL1, **c** 100% count EARL2, and **d** 50% count EARL2. Axial (left column), coronal (middle column), and sagittal views (right column) are shown

To evaluate the impact of count statistics (i.e. acquisition time), we split the original list-mode data of each whole body PET scan on an alternating count-wise basis into two new datasets, which were subsequently reconstructed into whole body images (as proposed in [21, 22]) using both EARL1 (4 mm) and EARL2 (4 mm + PSF) reconstructions. This generated two statistically equivalent but count-independent PET images each containing 50% of the original counts (referred to as split 1 and split 2). Due to the linear relationship between (decay corrected) the number of counts and the acquisition time, the whole body images reconstructed from split 50% count data served as surrogates for images acquired at 1.5 min per bed position (Fig. 1).

### Image analysis

All suspicious FDHT-avid lesions with uptake exceeding background were included. Volumes of interest (VOI) were delineated on the original PET images using a semi-automatic algorithm using a threshold of 50% of the peak value within the area of interest with correction for local background uptake [23]. From each VOI, we derived the average, peak, and maximum activity concentrations (AC; Bq/cc). Next, SUV was derived by dividing the tumour AC to a normalization factor. Two normalizations were used [12]: (a) injected dose per kg bodyweight (bw) and (b) area under the curve of the parent plasma calibrated image-derived input function (AUC-PP). The AR-positive tumour volume (ARTV; mL) was defined as the sum of all voxel volumes within a VOI. To assess lesion detectability, we generated a single voxel thick shell around the tumour VOI to determine local background activity, yielding the contrast-to-noise ratio (CNR) as follows:

2 $$ \mathrm{CNR}=\frac{\left({\mathrm{AC}}_{\mathrm{avg}}-{\mathrm{AC}}_{\mathrm{bgr}}\right)}{{\mathrm{SD}}_{\mathrm{bgr}}} $$

where AC_avg_ is the average tumour AC, AC_bgr_ is the average background AC, and SD_bgr_ is the standard deviation of AC in voxels included in the background shell.

To compare image noise levels of the 100% and 50% count images, a 3-cm diameter spherical VOI was placed in the liver, from which the coefficient of variation (COV%) was calculated as follows:

3 $$ \mathrm{COV}\%=\frac{{\mathrm{SD}}_{\mathrm{liver}}}{{\mathrm{AC}}_{\mathrm{liver}}}\times 100 $$

where SD_liver_ is the standard deviation of the ACs of voxels within the liver VOI and AC_liver_ is the mean AC of voxels within the liver VOI.

### Statistical analysis

**Fig. 2:**
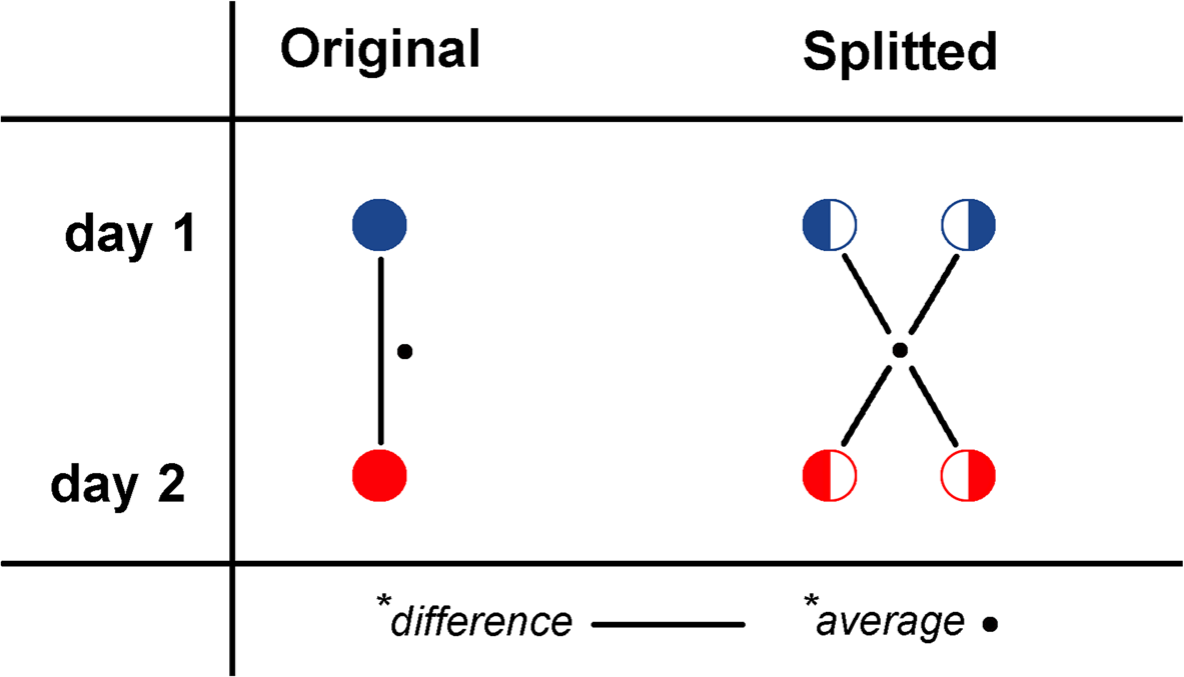
Schematic representation of assessment of test-retest variability of original 100% count scans and split 50% count scans, respectively. Note that, in contrast with original scans, split scans cannot be directly compared, as this would yield 4 individual comparisons underestimating true test-retest variability

Analyses were performed using SPSS statistics (v22, IBM) and Excel datasheets. Intrascan variability was defined as the difference in SUVs between the split scans of each original scan (e.g. split 1 vs split 2). Interscan variability was defined as the test-retest variability (repeatability) of SUVs. Both intra- and interscan variabilities were assessed on a per-lesion basis. Repeatability coefficients (RC%) were calculated from the standard deviations of the relative differences of measured SUVs between test-retest scans (day 1 vs day 2) and between split scans (split 1 vs split 2). To evaluate test-retest variability, 50% count scans were compared to the mean since split scans could not be directly compared as this would yield 4 comparisons (Fig. 2). Calculation of test-retest RCs was as follows (Eqs. 4–8):

4 $$ d=\frac{{\mathrm{SUV}}_2-{\mathrm{SUV}}_1}{\overline{{\mathrm{SUV}}_{\mathrm{orig}}}}\times 100 $$

5 $$ {d}_{i,j}=\frac{{\mathrm{SUV}}_{i,j}-\overline{{\mathrm{SUV}}_{i,j}}}{\overline{{\mathrm{SUV}}_{i,j}}}\times 100 $$

6 $$ \mathrm{SD}=\sqrt{\frac{\sum {\left(d-\overline{d}\right)}^2}{n-1}} $$

7 $$ {\mathrm{SD}}_{\mathrm{split}}=\sqrt{\frac{\sum {\left({d}_{i,j}-\overline{d_{i,j}}\right)}^2}{n-1}} $$

8 $$ \mathrm{RC}=\mathrm{SD}\times 1.96 $$

where *d* is the relative difference between day 1 (SUV_1_) and day 2 (SUV_2_) for original data, *d*_*i,j*_ is the relative difference of each split *i* (split 1 and split 2) on each day *j (*days 1 and 2) compared to the average SUV_*i*,*j*_; and SD is the standard deviation of relative test-retest differences (SD_split_ was scaled by factor 2 since SUVs were compared to the mean SUV_*i*,*j*_). RCs of intrascan variability were calculated using Eqs. 4, 6, and 8.

Bland-Altman plots with 95% limits of agreement, *R*^2^, and intraclass correlation coefficients (ICC) were calculated to assess inter- and intrascan variability [24]. ICCs represent the fraction of the total variability attributable to between lesion variability and were calculated using a two-way mixed model with absolute agreement definition [25]. To test for differences in repeatability between 100% and 50% count scans, we used a Wilcoxon signed-rank test designed to compare variances of dependent data (*p* < 0.05) [26].

## Results

Fourteen patients with a median age of 65 (IQR 47–75) years were included. Median Gleason score was 8 (IQR 5–10), and median PSA at imaging was 103 (IQR 11–1602) ng/mL. Median injected dosages of [^18^F]FDHT on day 1 and day 2 were 194 MBq (range 152–216) and 193 MBq (range 186–215) with residual activity in syringes/tubes of 37 MBq (range 26–63) and 36 MBq (18–54), respectively [12]. In two patients, no FDHT-avid lesions were detected. In the remaining 12 patients, 336 FDHT-avid lesions were visually detectable on both test and retest PET-CT scans.

### Image noise

**Fig. 3:**
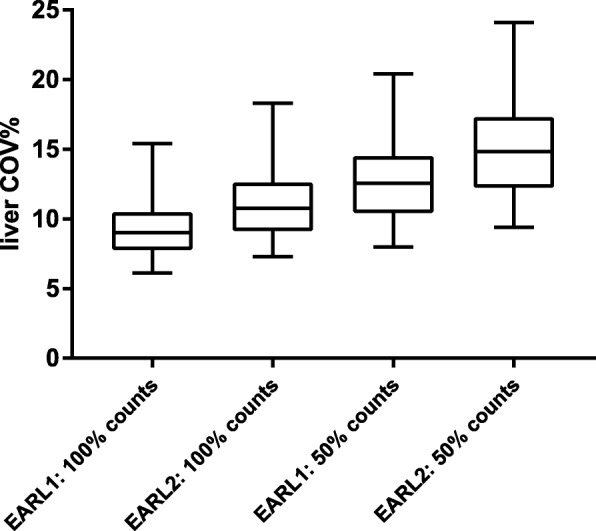
Liver COV% for 100% count and 50% count EARL1 and EARL2 images

Liver COV% of EARL1 images increased from a median 9.0 (IQR 7.9–10.4) to 12.5 (IQR 10.5–14.5) after count reduction (Fig. 3). For EARL2 images, liver COV% increased from a median 10.8 (IQR 9.2–12.4) to 13.2 (IQR 12.4–17.3) after count reduction.

### Semi-quantitative measurements

**Fig. 4:**
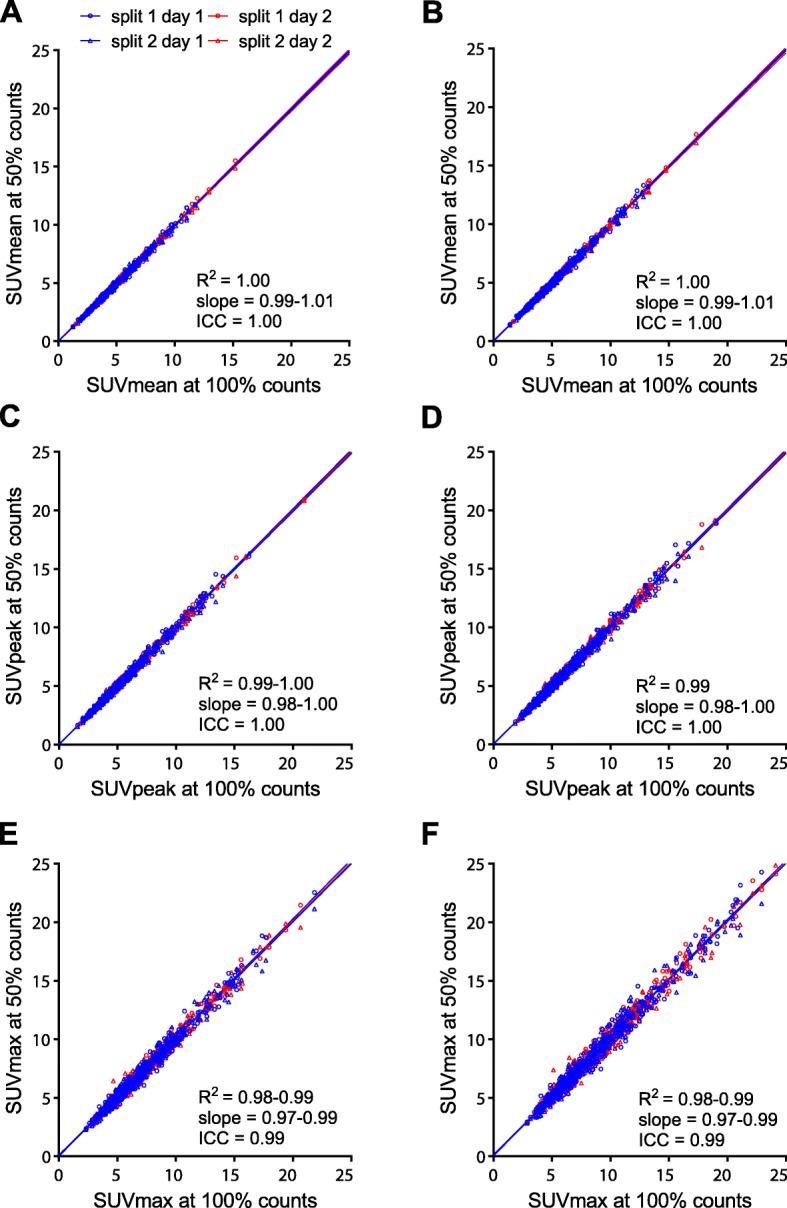
Correlations between SUVbw of original 100% count scans and split 50% count scans for SUVmean (**a, b**), SUVpeak (**c**, **d**), and SUVmax (**e**, **f**). Results from both EARL1 images (**a**, **c**, **e**) and EARL2 images (**b**, **d**, **f**) are shown

**Fig. 5:**
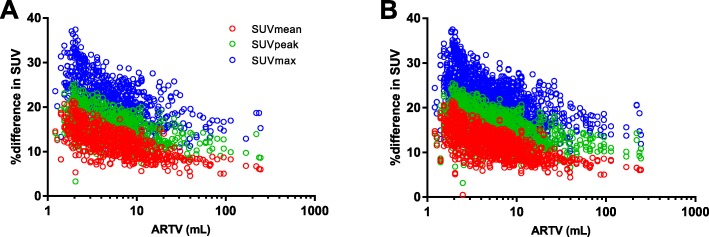
Relative difference (%) between SUVs derived from EARL1 images compared to SUVs derived from EARL2 images as a function of lesion ARTV. **a** Results from original (100% of counts) scans and **b** split (50% of counts) images

On EARL1 images, SUVmean, SUVpeak, and SUVmax of 100% count scans were highly correlated with those of 50% count scans (Fig. 4), with SUVmax being most affected by count reduction (albeit still with *R*^2^ > 0.98 and ICC = 0.99–1.00). Similar results were observed for EARL2 images (Fig. 4), with again SUVmax being most affected by count reduction (*R*^2^ > 0.98 and ICC = 0.99–1.00). On 100% count EARL2 images, SUVmean, SUVpeak, and SUVmax were median 12.1% (IQR 9.8–14.2%), 15.6% (IQR 13.1–17.9%), and 21.7% (IQR 18.4–25.1%) higher compared to EARL1 images. Similarly, on 50% count EARL2 images, SUVmean, SUVpeak, and SUVmax were a median 12.0% (9.6–14.2%), 15.5% (IQR 13.2–17.9%), and 21.6% (IQR 18.5–25.1%) higher compared to EARL1 images. These relative differences were inversely related to lesion ARTV (Fig. 5).

### Intrascan variability

**Fig. 6:**
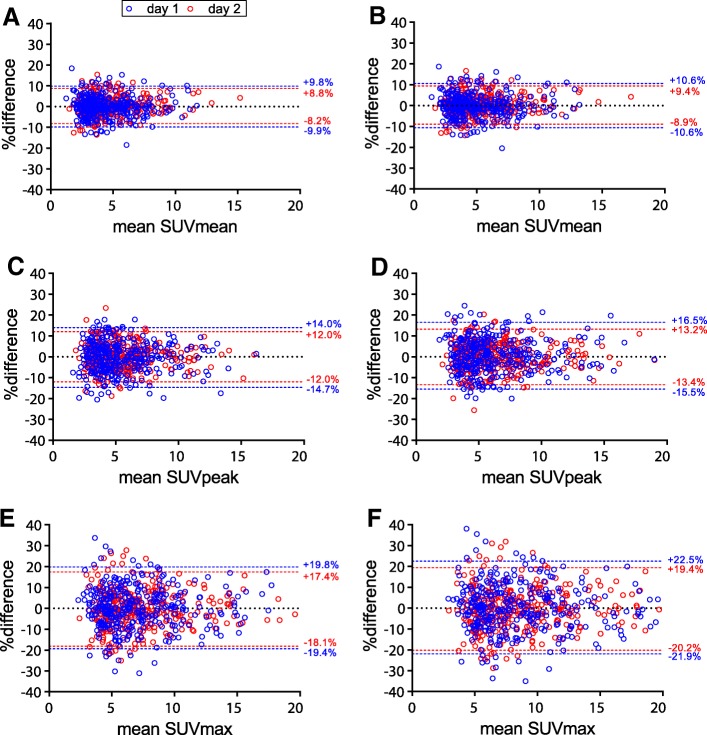
Bland-Altman graph of intrascan variability due to 50% count reduction for SUVmean (**a**, **b**), SUVpeak (**c**, **d**), and SUVmax (**e**, **f**). Results from both EARL1 images (**a**, **c**, **e**) and EARL2 images (**b**, **d**, **f**) are shown. Variability was derived from the relative difference in SUV between splits 1 and 2 of each scan on each day. Note that bw or AUC-PP normalization are not reported separately since normalization factors are identical for split 1 and split 2

RCs between 50% count scans on days 1 and 2, respectively, were 9.9% and 8.5% for SUVmean, 14.3% and 12.0% for SUVpeak, and 19.6% and 17.8% for SUVmax (Fig. 6) on EARL1 images. ICCs and *R*^2^ values between SUVs of 50% count EARL1 scans were high (ICC = 0.97–1.00; *R*^2^ = 0.95–0.99). On EARL2 images, RCs between 50% count scans on days 1 and 2, respectively, were 10.6% and 9.1% for SUVmean, 16.0% and 13.3% for SUVpeak, and 22.2% and 19.8% for SUVmax (Fig. 6). For EARL2 images, ICCs and *R*^2^ between SUVs of 50% count scans were almost identical to EARL1 (ICC = 0.97–0.99; *R*^2^ = 0.94–0.99.). SUV intrascan variability was volume-dependent for both EARL1 and EARL2 images (Additional file 1: Figure S1).

### Interscan variability

**Fig. 7:**
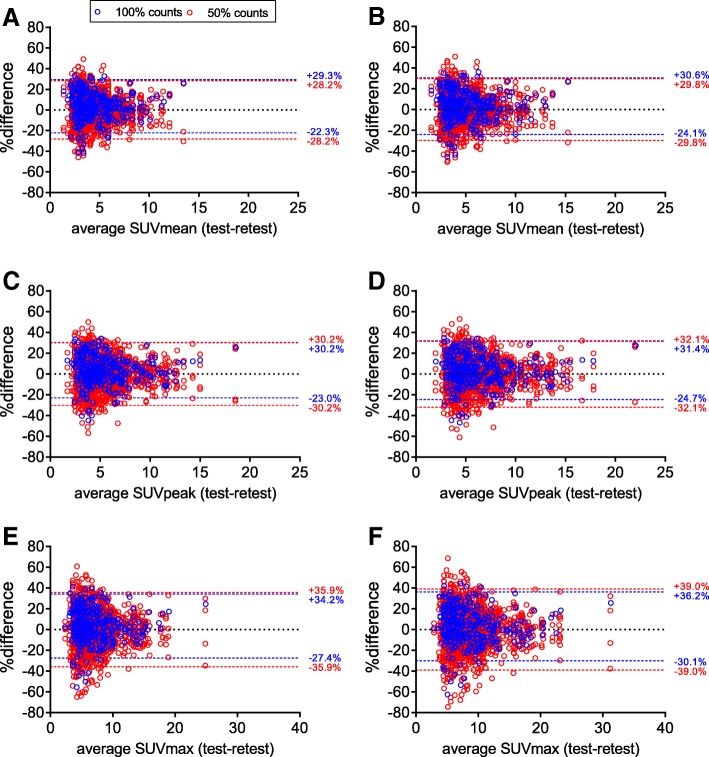
Bland-Altman graph of interscan (test-retest) variability of SUVmean (**a**, **b**), SUVpeak (**c**, **d**), and SUVmax (**e**, **f**) normalized to bodyweight at 100% and 50% of counts. Results from both EARL1 images (**a**, **c**, **e**) and EARL2 images (**b**, **d**, **f**) are shown

For EARL1 images, RCs of 50% count scans were higher than RCs of 100% count scans, but differences in variances were not significant (Fig. 7 and Table 1; ICC 0.94–0.97). A similar effect of count reduction on RCs was observed for selected lesions with ARTV > 4.2 mL, but in general, RCs for lesions > 4.2 mL were lower (Table 1). Repeatability of EARL2 was worse than EARL1 at both 100% and 50% count data (Fig. 6 and Table 1). Repeatability of EARL2 images was more affected by count reduction than EARL1 images, yet differences between variances of 100% and 50% count data were not significant (*p* = 0.53–1.00; Table 1). Normalizing SUVs to bw (Fig. 7) resulted in lower RCs than normalizing to AUC-PP (Additional file 1: Figure S2).

**Table 1:** Interscan variability (test-retest). Agreement between SUVs from test-retest scans (day 1 vs day 2) for original (100% count) scans and split (50% count) scans of both EARL1 and EARL2 images

Image	SUV normalization	SUV type	100% of counts		50% of counts		*p* value (all lesions)	*p* value (> 4.2 mL)
			RC% (all lesions)	RC% (> 4.2 ml)	RC% (all lesions)	RC% (> 4.2 mL)		
EARL1	Bodyweight	SUVmean	25.8	23.2	28.2	25.0	0.18	0.80
		SUVpeak	26.6	24.7	30.2	27.9	0.13	0.97
		SUVmax	30.8	26.9	35.9	31.5	0.50	0.41
	AUC-PP	SUVmean	27.6	27.5	29.7	30.0	0.52	0.95
		SUVpeak	27.7	28.2	31.3	32.2	1.00	1.00
		SUVmax	31.7	30.4	36.8	35.8	0.42	0.31
EARL2	Bodyweight	SUVmean	27.3	24.4	29.8	26.2	0.58	0.91
		SUVpeak	28.1	26.0	32.1	29.6	0.70	0.99
		SUVmax	33.1	28.6	39.0	34.0	0.37	0.74
	AUC-PP	SUVmean	28.5	28.0	30.6	30.3	0.37	0.99
		SUVpeak	28.8	29.2	32.8	33.4	0.69	1.00
		SUVmax	33.6	31.6	39.5	37.7	0.56	0.53

*p* value for differences between variances of test-retest differences of 100% vs 50% count images

*RC%* repeatability coefficient

### Lesion detectability

In general, the impact of count reduction on lesion detectability was small, with a median 4.6% (IQR 1.2–8.7%) reduction in CNR from median 3.7 (IQR 3.1–4.3) to 3.5 (IQR 2.9–4.1) on EARL1 images after count reduction. For EARL2 images, there was a median 4.6% (IQR 1.2–8.7%) reduction in CNR from median 3.9 (IQR 3.1–4.7) to 3.7 (IQR 2.9–4.5) after count reduction.

## Discussion

We investigated how accuracy, precision, and lesion detectability of analogue whole body [^18^F]FDHT PET-CT are affected by image count statistics and reconstruction protocol, to optimize imaging protocols for research and clinical use. Reducing counts by 50% introduced < 20% SUV intrascan variability for EARL1 images, which only increased test-retest variability to a small extent. Improving image spatial resolution by adhering to EARL2 guidelines might reduce the size-dependent bias in SUV, but it hampers repeatability and increases sensitivity to count statistics. Lesion detectability is only slightly affected by reduced counts and only marginally increased by resolution modelling.

SUVs of 50% count scans correlated highly with SUVs of 100% count scans, indicating accuracy is preserved at lower count statistics. However, when comparing split scans directly, a variability in SUV ranging 8.5% (SUVmean EARL1) to 22.2% (SUVmax EARL2) was observed. Hence, while SUV accuracy is maintained at low counts, its precision might be hampered. Still, test-retest variability only increased to a small and non-significant extent, which indicates that the statistical Poisson image noise is a minor determinant of SUV repeatability for [^18^F]FDHT.

SUV repeatability of oncological ^18^F-tracers (i.e. [^18^F]FDG, [^18^F]-fluorothymidine, [^18^F]-fluoromethylcholine, [^18^F]FDHT) ranges between 10 and 30%, yielding 30% as the preferred upper threshold for SUV variability for use in e.g. response monitoring studies [27–30]. As expected, repeatability of SUVmax was most affected by count reduction and EARL2 reconstruction, yielding RCs > 30%. In contrast, SUVpeak seemed to be robust to both count statistics and reconstruction protocol, yielding an RC of approximately 30% after count reduction, which was even lower (27.9%) when only lesions > 4.2 mL were considered. The improved repeatability of SUV when excluding small lesions seems a direct consequence of the size dependency of intrascan variability at reduced counts (Additional file 1: Figure S1). Note that test-retest variability of [^18^F]FDHT can be even lower when evaluating only selected target lesions, or analysing on a patient instead of lesion basis [12]. In the current study, all avid lesions were primarily included to avoid selection bias and also evaluate the effect of count reduction on smaller and less avid lesions.

Between SUV normalizations, differences in test-retest variability were observed, with larger variability in SUVauc-pp (> 30%) compared SUVbw. While SUV normalized to AUC-PP correlates better with reference pharmacokinetic parameters than SUV normalized to bodyweight [19], deriving it is more technically demanding and less precise compared to more simple factors such as dose per bodyweight, making it less suitable for multicentre studies. Hence, a trade-off between accuracy, precision, and ease of use has to be made when selecting the preferred SUV normalization. For example, while SUVpeak normalized to bodyweight had a RC of 30% at half of counts, it exceeded 30% when normalizing to AUC-PP rendering it unfit for response assessment.

Partial-volume effects generally result in volume-dependent underestimations of tumour SUV and possibly hamper lesion detectability [31]. Correcting for PVE in the reconstruction algorithms might be particularly important in [^18^F]FDHT due to the high frequency of small (e.g. < 4.2 mL) detected lesions. Novel reconstruction algorithms incorporating the PSF either within or after reconstruction have been proposed to improve image resolution [17]. The EARL2 standards have adopted these algorithms as a step forward in scanner calibration harmonization between centres [15]. However, PSF reconstructions are known to suffer from noise propagation and image artefacts (e.g. Gibbs phenomenon resulting in edge overshoot), which might lead to misinterpretation regarding treatment effects [17, 18, 32]. Indeed, we observed that repeatability was worse for the EARL2 reconstruction with higher sensitivity to count statistics, resulting in a higher minimal detectable change for response assessment.

Previous reports argued that PSF reconstructions should be used for qualitative purposes (i.e. lesion detection) and that non-PSF images (such as EARL1) should be used for tumour quantification [18, 33]. However, Quak et al. found that with additional image filtering the higher lesion detection and image resolution of PSF images do not need to be impaired in order to meet the EARL criteria [34]. In the present study, we observed a very small increase in lesion CNR when PSF was applied. This will not likely result in clinically relevant different conclusions regarding the extent of disease or intrapatient heterogeneity (Fig. 1) due to the vast amount of detected lesions (336 lesions in 12 patients). The small reduction in CNR by < 5% after count reduction is also not likely to have clinical consequences (Fig. 1). This corresponds to [^18^F]FDG PET-CT data in several cancer types, where reducing acquisition time from 3 to 1.5 min per bed position reduced image quality, but did not impair lesion detection rates [13].

Another factor affecting image count statistics is the injected tracer dosage. In the present cohort, patients received a relatively low dosage compared to other cohorts from the recent multicentre study [12]. However, while SUV test-retest variability varied between centres, the authors did not observe a direct relationship between injected dosage and repeatability [12]. This might be explained by differences in other factors determining repeatability, such as the observer variability in tumour delineation, PET system specifics, adherence to imaging protocols (i.e. uptake interval), and methods for acquiring the SUV normalization factors. Hence, count statistics did not appear to be the main determinant of [^18^F]FDHT repeatability, which we confirm in the current study where non-significant increases in test-retest RCs were observed after count reduction. Therefore, a potentially modifiable and important determinant of SUV variability in [^18^F]FDHT imaging seems to be the choice of normalization factors, which, again, need some trade-off between accuracy and precision to be made.

The present study contains several limitations. First, while splitting data on a count-wise basis enables evaluation of Poisson noise induced by count reduction, the 50% count scans do not fully represent a 50% shorter image acquisition. However, [^18^F]FDHT kinetics commonly reach a plateau after 20–30 min, yielding stable SUV during the whole body acquisition [8]. Second, the present study contains data acquired on a PET system of a single vendor. As between vendors the overlap between bed positions differs, count reduction might have a different impact on measurement variability for these PET systems. Also, for novel PET systems, which may have higher sensitivities and better time-of-flight performance, in particular for the new digital systems, the impact of reducing acquisition times on measurement variability will be even smaller. Hence, for these systems acquisition times may be reduced even further, but this remains to be investigated for each type of system. As investigated in the present study for analogue PET, a reduction up to 50% compared with the current standard practice seems to be feasible for diagnostic and response assessment purposes, given that the use of SUVmax is avoided.

The current approach for evaluating the sensitivity of whole body PET-CT acquisition to scan statistics can be extended to other tracers currently being investigated and/or implemented in clinical practice, such as PSMA-ligand PET-CT. For adequate evaluation of these tracers, however, test-retest data should be available.

## Conclusion

In [^18^F]FDHT PET-CT studies, noise-induced SUV variability leads to small increases in test-retest variability, which improves when excluding small lesions. Novel EARL2-compliant reconstruction increases lesion SUVs and marginally increases CNRs. However, it requires higher count statistics to preserve adequate precision. For SUVpeak normalized to bodyweight, test-retest variability remained below 30% when lesions < 4.2 mL were excluded, which is generally acceptable for oncological [^18^F]-tracers. In contrast, SUVmax was substantially affected by count reduction and EARL2 reconstruction; hence, its use should be avoided. Lesion detectability is only slightly impaired by reducing counts by 50%, which might not be clinically relevant in the mCRPC population.

Taken together, with the current imaging procedure on an analogue PET-CT system, count statistics are more than sufficient and could even be reduced by 50% without affecting diagnostic performance and at a small expense of reduced precision of response assessments. Acquisition time reduction is feasible for staging and response assessment purposes.

## Additional file

Additional file 1: **Figure S1.** Intrascan variability due to 50% count reduction as a function of lesion ARTV for SUVmean (A and B), SUVpeak (C and D), and SUVmax (E and F). Results from both EARL1 images (A, C, E) and EARL2 images (B, D, F) are shown, with limits of agreement from Bland-Altman analyses. Figure S2 Bland-Altman graph of interscan (test-retest) variability of SUVmean (A and B), SUVpeak (C and D), and SUVmax (E and F) normalized to AUC-PP at 100% and 50% of counts. Results from both EARL1 images (left column) and EARL2 images (right column) are shown. (PDF 1080 kb)

## Data Availability

Data are available on request to the corresponding author.

## Abbreviations

[^18^F]FDHT: ^18^F-fluorodihydrotestosterone
AC: Activity concentration
AR: Androgen receptor
ARSi: AR signalling inhibitor
ARTV: AR-positive tumour volume
AUC-PP: Parent plasma area under the curve
BLOB-OS-TF: Iterative time-of-flight reconstruction algorithm
bw: Bodyweight
CNR: Contrast-to-noise ratio
COV%: Coefficient of variation
EANM: European Association of Nuclear Medicine
EARL: EANM Research Ltd.
ICC: Intraclass correlation coefficient
mCRPC: Metastatic castration-resistant prostate cancer
PET-CT: Positron emission tomography
PSF: Point spread function
RC: Repeatability coefficient
SUV: Standardized uptake value
VOI: Volume of interest
