# Supplementary material for: Sensitivity of 18F-fluorodihydrotestosterone PET-CT to count statistics and reconstruction protocol in metastatic castration-resistant prostate cancer
Source: EJNMMI Res. 2019 Jul 30;9:70. doi: 10.1186/s13550-019-0531-8 (PMC6667590; doi:10.1186/s13550-019-0531-8)
Supplement: Supplementary file 1 — Figure S1. Intrascan variability due to 50% count reduction as a function of lesion ARTV for SUVmean (A and B), SUVpeak (C and D), and SUVmax (E and F). Results from both EARL1 images (A, C, E) and EARL2 images (B, D, F) are shown, with limits of agreement from Bland-Altman analyses. Figure S2 Bland-Altman graph of interscan (test-retest) variability of SUVmean (A and B), SUVpeak (C and D), and SUVmax (E and F) normalized to AUC-PP at 100% and 50% of counts. Results from both EARL1 images (left column) and EARL2 images (right column) are shown. (PDF 1080 kb) [file 13550_2019_531_MOESM1_ESM.pdf]

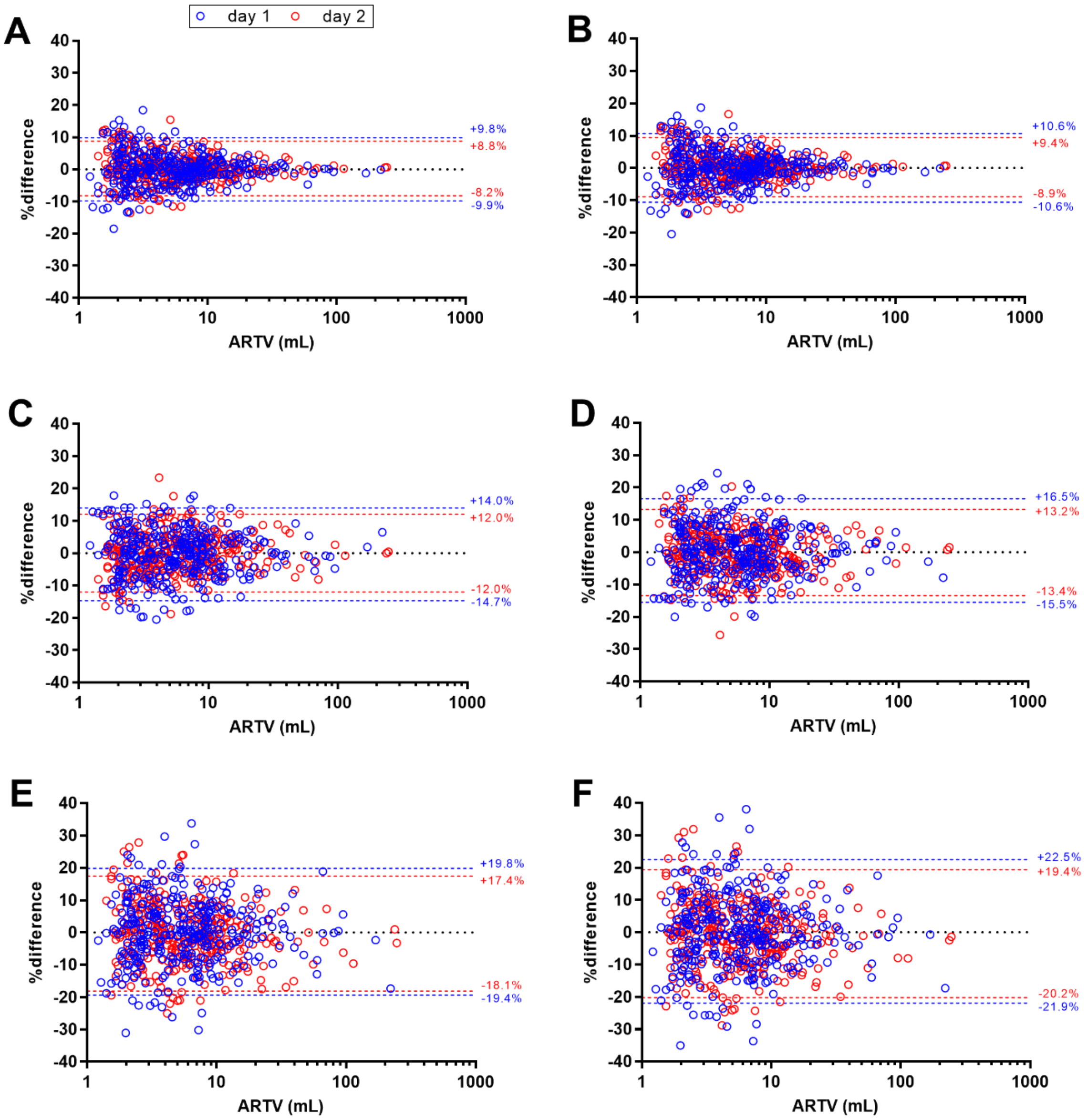

Supplemental Figure 1) Intrascan variability due to 50% count reduction as function of lesion ARTV for SUVmean (A and B), SUVpeak (C and D), and SUVmax (E and F). Results from both EARL1 images (A, C, E) and EARL2 images (B, D, F) are shown, with limits of agreement from Bland-Altman analyses.

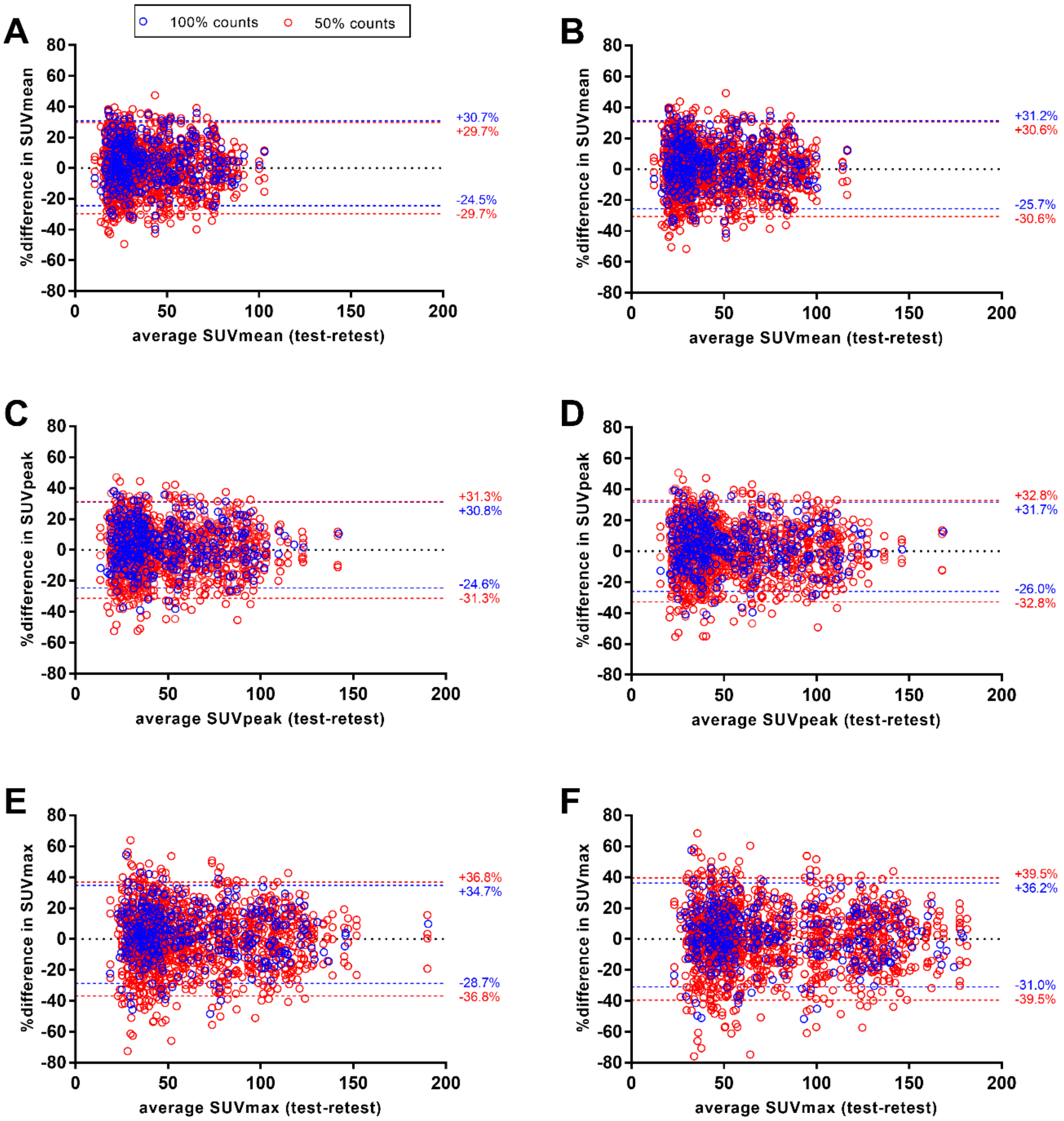

Supplemental Figure 2) Bland-Altman graph of interscan (test-retest) variability of SUVmean (A and B), SUVpeak (C and D), and SUVmax (E and F) normalized to AUC-PP at 100% and 50% of counts. Results from both EARL1 images (left column) and EARL2 images (right column) are shown.
